# Glucose metabolism after distal pancreatectomy - deterioration of beta cell function becomes noticeable at an early stage: a retrospective cohort study

**DOI:** 10.1186/s12893-025-02867-z

**Published:** 2025-04-09

**Authors:** Mikheil Kalandarishvili, Florian Oehme, Olga Radulova-Mauersberger, Nicole Kipke, Michele Solimena, Christian Teske, Nicolas Mibelli, Jürgen Weitz, Marius Distler, Sebastian Hempel

**Affiliations:** 1 Department of Visceral, Thoracic and Vascular Surgery, University Hospital and Faculty of Medicine Carl Gustav Carus, Technische Universität Dresden, Fetscherstrasse 74, 01307 Dresden, Germany; 2 Paul Langerhans Institute Dresden of the Helmholtz Center Munich at University Hospital and Faculty of Medicine Carl Gustav Carus, Technische Universität Dresden, Dresden, Germany; 3 Molecular Diabetology, University Hospital and Faculty of Medicine, Technische Universität Dresden, Dresden, Germany; 4 German Center for Diabetes Research (DZD e.V), Neuherberg, Germany

**Keywords:** Distal pancreatectomy, Blood glucose homeostasis, Postoperative hyperglycemia, New-onset diabetes mellitus, Pancreatogenic diabetes

## Abstract

**Background:**

Distal pancreatectomy (DP) can worsen pancreatic endocrine function. Effects on glucose metabolism and underlying mechanisms after DP remains a topic of significant interest and not yet fully understood. This study aimed to examine the impact of DP on blood glucose homeostasis with a particular focus on metabolic outcomes and development of postoperative diabetes.

**Methods:**

Considered were all patients who underwent DP between 01/2010 and 09/2021 and participated simultaneously in extended blood glucose monitoring with a 12 months follow-up. Blood samples were analyzed for markers of pancreatic endocrine function both fasting and after an oral glucose tolerance test preoperatively and 3 and 12 months after DP.

**Results:**

Included patients (*n* = 69) were preoperatively categorized into three groups according to American Diabetes Association (ADA) criteria: 17 patients (24.6%) were normoglycemic (NG), 22 (31.9%) had prediabetes (impaired fasting glucose / impaired glucose tolerance – IFG/IGT) and 30 (43.5%) had diabetes mellitus (DM). In the NG subgroup, beta-cell function (HOMA2%B - updated homeostasis model assessment) significantly decreased from 117.4% (101.1–135%) to 66.9% (49.7–102.1%) at 12 months postoperatively (*p* < 0.05). Insulin sensitivity (HOMA2%S) significantly increased from 48.2% (33.4–66.9%) to 63.5% (49.8–86%) at 12 months postoperatively (*p* < 0.05). In the IFG/IGT subgroup, there was a non-significant trend of decreased HOMA2%B and increased HOMA2%S postoperatively. Postoperatively, 11.8% of NG patients and 60% of prediabetic patients developed DM.

**Conclusion:**

DP already leads to significant changes in glucose metabolism within a 12 month follow-up period. Patients with preoperative prediabetes are particularly at high risk of developing postoperative DM. Therefore, the indication for DP should be critically evaluated, especially in cases with a relative indication for surgery. If possible parenchymal sparing surgical options should be contemplated.

**Trial registration:**

Not applicable.

## Introduction

Distal pancreatectomy (DP) is utilized in the management of various pancreatic pathologies, including benign and malignant tumors, cysts and chronic pancreatitis (CP). With DP, the likelihood of developing new-onset diabetes mellitus (NODM) is significantly higher than with pancreaticoduodenectomy (PD). The incidence of NODM after DP varies between 7 and 51% [1] compared to 18–39% after PD [2]. Postoperative hyperglycemia and the development of NODM are recognized complications following DP, yet the mechanisms driving these phenomena and strategies for their prevention and management remain areas of active investigation.

Currently, there are two common classifications of diabetes, according to the World Health Organization (WHO) and the American Diabetes Association (ADA). Pancreatectomy-associated diabetes is classified under the group “Specific types of diabetes due to other causes” (ADA) or “Other specific types of diabetes” (WHO), and specifically under the subgroup “diseases of the exocrine pancreas” [3, 4]. The timeframe defining postoperative DM as pancreatectomy-associated is not precisely defined. However, the cause is described as the reduction in the total number of beta cells due to resection, resulting in a consequent reduction in insulin production [5]. Furthermore, according to a systematic review by De Bruijn NODM is not always insulin-dependent, and the necessity of insulin therapy varies among patients after DP between 36 and 100% [1].

Most existing studies investigating NODM after DP attributed to a consecutive reduction in beta-cell count. Extended metabolic investigations are often missing. Therefore, in this study, we aim to contribute further evidence on the effects of DP on blood glucose metabolism.

## Methods

### Study design

This study was conducted as a retrospective study, observing and evaluating data collected prospectively as part of extended blood glucose monitoring protocol within the OGTT-study offered to all patients undergoing partial pancreatectomy in our hospital since 2010. The performance of pre- and post-surgical oral glucose tolerance tests (OGTT) within these OGTT-study was approved by the local ethics committee of the Technische Universität Dresden (TUD) (approval number EK 151062008). Medical records including pre- and postoperative data as well as follow-up data for each case were obtained from a prospective database and retrospectively analyzed. The experimental protocol of this retrospective analysis was separately approved by the local ethics committee of the TUD (approval number EK-174042022). All methods were carried out in accordance with relevant guidelines, and written informed consent was obtained from all included patients.

### Patient cohort

**Fig. 1:**
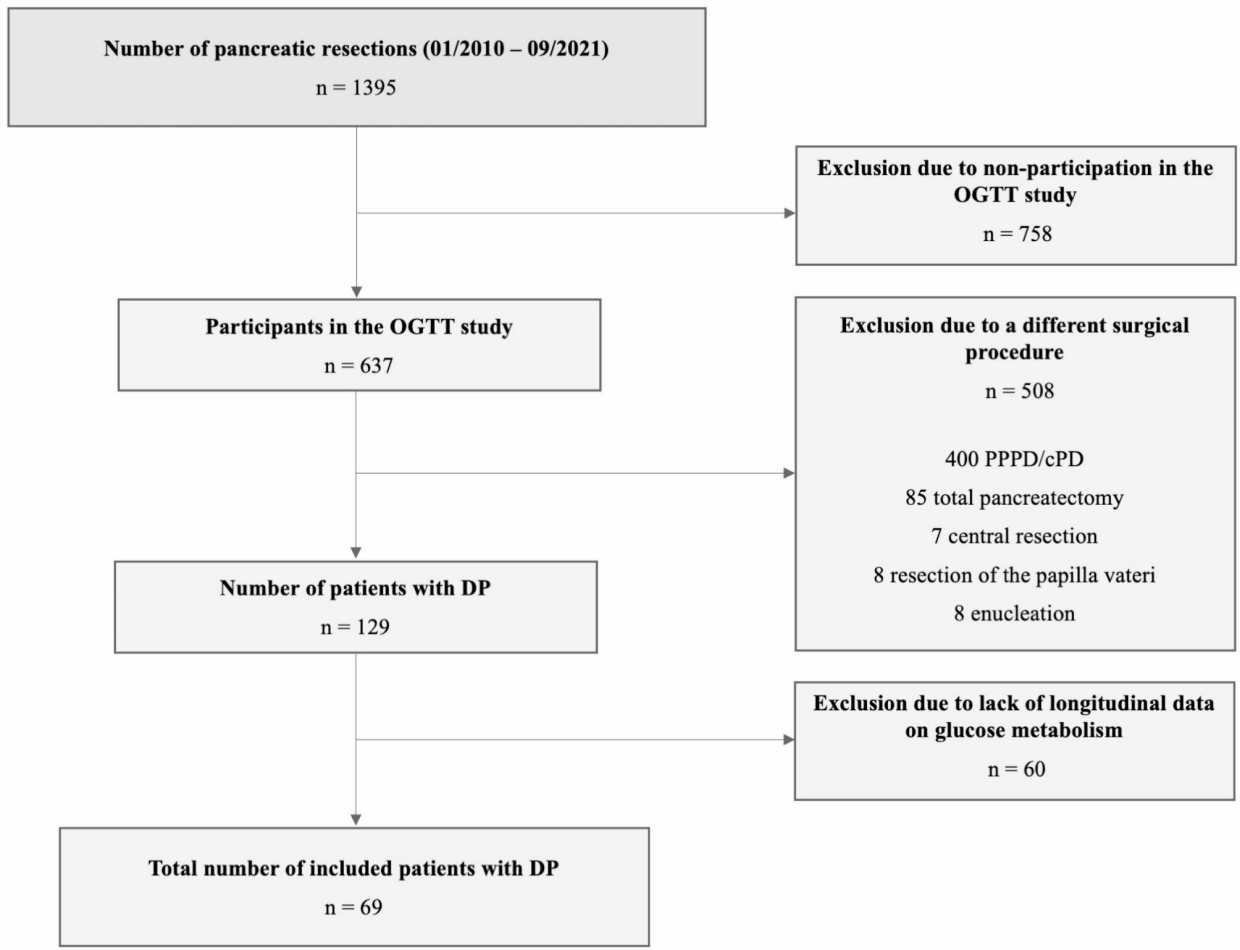
Flowchart of the included patients Abbreviations: cPD – classic pancreaticoduodenectomy (Whipple procedure); DP – distal pancreatectomy; OGTT – oral glucose tolerance test; PPPD – pylorus-preserving pancreaticoduodenectomy

A total of 1395 pancreatic resections were performed at the Department of Visceral-, Thoracic and Vascular Surgery, University Hospital Carl Gustav Carus Dresden, Technische Universität Dresden, Germany between January 2010 and September 2021 for various indications. Of all patients undergoing elective partial pancreatectomy 637 participated in our OGTT-study during the observational period, including 129 who underwent DP. Among these, 60 had to be excluded from our analysis due to missing postoperative laboratory follow-up. Ultimately, 69 patients were considered for further analysis in this study (Fig. 1).

### Blood glucose homeostasis analysis

All included patients underwent comprehensive blood analysis preoperatively, as well as at 3 and 12 months postoperatively, including the measurement of fasting glucose, HbA1c, C-peptide, proinsulin, and insulin levels. These parameters were assessed both in the fasting state and during the OGTT. The OGTT was omitted in patients with known diabetes or contraindications, including pathological findings (fasting hyperglycemia ≥ 126 mg/dL or ≥ 7 mmol/L; occasional hyperglycemia ≥ 200 mg/dL or ≥ 11.1 mmol/L; HbA1c ≥ 6.5% or ≥ 48 mmol/mol), treatment with antidiabetic medication, endocrine disorders (e.g., Cushing’s disease, pheochromocytoma, acromegaly), altered gastrointestinal passage, disturbances in potassium or magnesium levels, and liver insufficiency.

The OGTT was performed after a fasting period of 10–16 h following at least three days of a diet containing more than 150 g of carbohydrates per day. First, fasting blood glucose was measured, along with insulin, proinsulin and C-peptide. Patients then consumed 75 g of glucose monohydrate dissolved in approximately 150–200 mL of water within 5 min. Blood samples were collected while patients remained in a seated or lying position without prior physical exertion. Smoking was prohibited before and during the test, and patients had to remain inactive for 2 h. Additional blood samples were taken at 60 and 120 min to measure glucose and, if required, the aforementioned parameters.

Based on the preoperative results of these blood tests, the patient cohort was categorized into three groups according to ADA criteria [3]: normoglycemic (NG), patients with prediabetes (impaired fasting glucose / impaired glucose tolerance - IFG/IGT) and patients with diabetes mellitus (DM). Markers of pancreatic endocrine function including steady-state beta-cell function (%B), insulin resistance (IR), and insulin sensitivity (%S) were determined according to the updated Homeostasis Model Assessment (HOMA2) using the homacalculator [6]. This requires simultaneous measurement of glucose and insulin or C-peptide after at least 12 h of fasting. In this study, the HOMA2 indices were calculated using glucose and C-peptide values based on the formula by Levy et al. [6]. All patients were negative for the presence of circulating autoantibodies directed against type 1 diabetes autoantigens GAD65, insulin, IA-2, and Znt8, which were measured as described [7].

### Surgical technique

At our institution, DP is primarily performed using minimally invasive techniques, either robotic-assisted or laparoscopic, by a team of several experienced pancreatic surgeons. In patients with locally advanced disease and before establishment of robotic surgery, conventional open surgery was often preferred. For the conventional approach, either a left-sided upper abdominal transverse laparotomy or a midline laparotomy was performed, depending on the surgeon´s preference. In all included cases, the transection line of the pancreas was defind at the level of the mesentericoportal axis. Both in the conventional or minimally invasive approaches, pancreatic transection was performed using either a stapler or a scalpel, followed by oversewing of the pancreatic duct and fish-mouth closure of the pancreatic remnant. The pancreatic stump was covered with a falciform ligament patch.

When technically feasible and in the absence of malignancy, spleen-preserving procedures were performed. At the end of the operation, a lavage drain was placed near the pancreatic stump.

### Study endpoints

The primary endpoint was to assess the effect of distal pancreatectomy on glucose metabolism. In addition to HbA1c, all the aforementioned parameters were also examined at 60 and 120 min following glucose provocation as part of the OGTT. To further understand the residual beta-cell function, insulin resistance, and insulin sensitivity after surgery, the relevant HOMA indices within the subgroups were calculated and illustrated. The secondary endpoints included the assessment of the incidence and therapy of NODM after DP.

### Statistical analysis

The statistical analysis underlying this study was conducted using SPSS Version 26 (IBM Corp, Armonk NY, USA). Normality testing of continuous data was performed using the Kolmogorov-Smirnov test along with graphical distribution analysis. Variance homogeneity analyses were conducted using Levene’s test and were considered as prerequisite analyses. Graphical initial analyses ensured understanding of data distribution and quality. Descriptive statistical analysis was conducted regarding the distribution of individual categories concerning patients’ baseline characteristics, perioperative data, and the occurrence of peri- and postoperative complications. Presentation of continuous variables, such as parameters for assessing blood sugar homeostasis, duration of hospital stay and follow-up parameters of blood sugar homeostasis at 3 and 12 months, was realized using median and interquartile range (IQR).

ANOVA analysis was employed to compare clinical characteristics and blood sugar homeostasis among groups (NG, IGT, and DM) and among time points (preoperative, 3 and 12 months postoperatively). Pairwise comparisons among time points required repeated measures analyses. A generalized linear model was applied and Bonferroni correction (repeated measures ANOVA) ensured reduction of Type I error. Mauchly’s sphericity was tested prior to analysis. *P* < 0.05 was considered statistically significant. Inclusion criteria for analysis were complete datasets for each patient. Patients with missing data were treated as missing completely at random and excluded from further analyses.

## Results

### Baseline patient characteristics

In total, 69 patients were included in the study and divided into 3 subgroups according ADA: NG (*n* = 17; 24.6%), IFG/IGT (*n* = 22; 31.9%) and DM (*n* = 30; 43.5%). The median age was 65 years (IQR 56–73) with the female predominance (55.1%). The median body mass index (BMI) was 25.8 kg/m^2^ (IQR 23.3–29). In the cohort, 42 patients (60.9%) presented with arterial hypertension (HTN), 17 patients (24.6%) with chronic kidney disease (CKD), 14 patients (20.3%) with chronic heart failure (CHF), and 9 patients (13%) with coronary heart disease (CHD). Interestingly, these comorbidities were more frequently observed in diabetic patients compared to non-diabetic and pre-diabetic individuals. Notably, HTN and CHF were statistically significantly more prevalent in diabetic individuals (< 0.01 and *p* < 0.05, respectively). Preoperatively, 9 patients (30%) among the diabetics were treated with insulin, 8 patients (26.6%) with oral hypoglycemic agents (OHA) and 5 patients (16.6%) with combination therapy (insulin and OHA). Additionally, 8 patients (26.6%) with DM received no antidiabetic medication and were managed with diet alone. Most patients, comprising 35 individuals (50.7%), were categorized under ASA class 2. Table 1 displays all baseline patient characteristics.

**Table 1:** Baseline patient characteristics

Variable	Overall	ND	IFG/IGT	DM	*p*-Value *
Patients [*n*] (%)	69	17 (24.6)	22 (31.9)	30 (43.5)	
Sex [*n*] (%)					
M W	31 (44.8) 38 (55.1)	5 (29.4) 12 (70.6)	9 (40.9) 13 (59.1)	17 (56.7) 13 (43.3)	0.18
Median age [years] (IQR)	65 (56–73)	58 (42–68)	62 (56.8–70.3)	71 (58.8–78)	**< 0.01**
Median BMI [kg/m²] (IQR)	25.8 (23.3–29)	23.4 (21.4–25.4)	25.5 (23.8–28.1)	27 (25.1–30.4)	**0.02**
Smoking [*n*] (%)	12 (17.4)	6 (35.3)	4 (18.2)	2 (6.7)	**< 0.05**
Alcohol [*n*] (%)	5 (7.2)	2 (11.8)	3 (13.6)	0	0.12
Weight loss [*n*] (%)	24 (34.8)	6 (35.3)	5 (22.7)	13 (43.3)	0.3
HTN [*n*] (%)	42 (60.9)	6 (35.3)	12 (54.5)	24 (80)	**< 0.01**
CHF [*n*] (%)	14 (20.3)	3 (17.6)	1 (4.5)	10 (33.3)	**0.04**
CHD [*n*] (%)	9 (13)	0	2 (9.1)	7 (23.3)	0.06
CKD [*n*] (%)	17 (24.6)	2 (11.8)	4 (18.2)	11 (36.7)	0.11
IDDM [*n*] (%)	14 (20.3)	0	0	14 (46.7)	n.a.
OHA [*n*] (%)	13 (18.8)	0	0	13 (43.3)	n.a.
ASA [*n*] (%)					
ASA 1 ASA 2 ASA 3 ASA 4	6 (8.7) 35 (50.7) 27 (39.1) 1 (1.4)	3 (17.6) 10 (58.8) 4 (23.5) 0	2 (9.1) 14 (63.6) 6 (27.3) 0	1 (3.3) 11 (36.7) 17 (56.7) 1 (3.3)	0.12

* One-way analysis of variance (ANOVA) with repeated measurements, *p*-value refers to analysis across all timepoints

Abbreviations: ASA - American Society of Anesthesiologists; BMI - Body Mass Index; CHD - coronary heart disease; CHF - chronic heart failure; CKD - chronic kidney disease; DM - diabetes mellitus; IDDM - insulin-dependent diabetes mellitus, IFG/IGT - impaired fasting glucose/impaired glucose tolerance; IQR - interquartile range; HTN - arterial hypertension; NG - normoglycemic; OHA - oral hypoglycemic agents

### Indications and surgical outcomes

Table 2 illustrates the histopathological findings and surgical outcomes of the patients. The study included patients with both benign and malignant diseases, including pancreatic ductal adenocarcinoma (PDAC) (*n* = 21; 30.4%), intraductal papillary mucinous neoplasm (IPMN) (*n* = 23; 33.3%), neuroendocrine tumors (NET) (*n* = 7; 10.1%), CP (*n* = 5; 7.2%) and pancreatic metastasis in the pancreatic tail region from other origin (*n* = 5; 7.2%). None of the neuroendocrine tumors were clinically hormone-active. The remaining 8 patients (11.6%) underwent surgery for a variety of conditions, including endometrial heterotopia (*n* = 1), postoperative pancreatic fistulas (POPF) after splenectomy (*n* = 1), symptomatic pseudocyst (*n* = 1), serous oligocystic adenoma in the pancreatic tail region (*n* = 2), inflammatory mass of the pancreatic corpus (*n* = 1), solid pseudopapillary neoplasm (*n* = 1) and mucinous cystic neoplasm of the pancreatic tail (*n* = 1). Relevant postoperative complications according to the Clavien-Dindo classification (CDC ≥ 3) were observed in 23 cases (33%). Out of them, 21 patients developed a POPF grade B (30.4%), and 1 patient developed a POPF grade C (1.4%). Among the 22 patients with clinically relevant POPF (grade B/C), 9 patients already exhibited a diabetic metabolic state preoperatively. Of the 13 patients in the NG and IFG/IGT groups, 7 developed NODM postoperatively. However, no statistical significance was observed. Postpancreatectomy hemorrhage (PPH) of varying degrees was observed in 5 patients (7.25%). Regarding postoperative complications and surgical outcome, there was no significant difference with respect to preoperative glycemic status. The overall median length of hospital stays was 12 days (IQR 9–17). Open surgery was performed in 42 patients (60.9%) and minimal-invasive surgery in 27 patients (39.1%) of which 12 patients underwent laparoscopic surgery and 15 patients underwent robot-assisted surgery. Simultaneous splenectomy was required in 53 patients (76.8%) and multivisceral resection was performed in 12 cases (17.4%). The median operative time was 231 min (IQR 166–279 min). No patient died during the hospital stay or within 30 days postoperatively.

**Table 2:** Indications and surgical outcome

Variable	Overall	NG (*n* = 17)	IFG/IGT (*n* = 22)	DM (*n* = 30)	*p*-Value*
Histopathological type					
PDAC [*n*] (%) NET [*n*] (%) IPMN [*n*] (%) Metastasis [*n*] (%) CP [*n*] (%) Other [*n*] (%)	21 (30.4) 7 (10.1) 23 (33.3) 5 (7.2) 5 (7.2) 8 (11.6)	4 (23.5) 1 (5.9) 8 (47.1) 1 (5.9) 1 (5.9) 2 (11.8)	3 (13.6) 3 (13.6) 8 (36.4) 2 (9.1) 2 (9.1) 4 (18.2)	14 (46.7) 3 (10) 7 (23.3) 2 (6.7) 2 (6.7) 2 (6.7)	0.52
Complications CDC ≥ 3 [*n*] (%)	23 (33)	4 (23.5)	9 (40.9)	10 (33.3)	0.52
POPF B/C [*n*] (%)	22 (31.8)	5 (22.7)	8 (36.4)	9 (40.9)	0.73
PPH [*n*] (%)	5 (7.25)	2 (40)	1 (20)	2 (40)	0.67
Length of hospital stay [days] (%)	12 (9–17)	9 (8-10.5)	12 (9.8–16.3)	13.5 (11-22.3)	**< 0.01**
Type of operation [*n*] (%)					
Open Laparoscopic Robot-assisted	42 (60.9) 12 (17.4) 15 (21.7)	5 (29.4) 3 (17.6) 9 (52.9)	13 (63.6) 4 (18.2) 4 (18.2)	23 (76.7) 5 (16.7) 2 (6.7)	**< 0.01**
Splenectomy [*n*] (%)	53 (76.8)	13 (76.5)	15 (68.2)	25 (83.3)	0.44
MVR [*n*] (%)	12 (17.4)	2 (11.8)	4 (18.2)	6 (20)	0.77
Median duration of operation [min] (IQR)	231 (166–279)	215 (173–245)	232 (178.3–279)	234 (148.8–294.5)	0.63
30-day mortality	0	0	0	0	n.a.
In-hospital mortality	0	0	0	0	n.a.

* One-way analysis of variance (ANOVA) with repeated measurements, *p*-value refers to analysis across all timepoints

Abbreviations: CDC – Clavien Dindo classifcation; CP - chronic pancreatitis; DM – diabetes mellitus; IFG/IGT – impaired fasting glucose/impaired glucose tolerance; IPMN - intraductal papillary mucinous neoplasm; IQR - interquartile range; MVR – multivisceral resection; NG – normoglycemic; NET - neuroendocrine tumor; PDAC - pancreatic ductal adenocarcinoma; POPF - postoperative pancreatic fistula; PPH - postpancreatectomy hemorrhage

### Effects of DP on blood glucose homeostasis in normoglycemic patients

The group of normoglycemic patients experienced a slight reduction in BMI during the first three months, which returned to baseline within a year. Patients in the NG subgroup exhibited normal glucose homeostasis preoperatively. Three and twelve months after surgery, fasting glucose (*p* < 0.01), HbA1c (*p* < 0.01) and insulin levels (*p* < 0.01) changed significantly compared to preoperative levels. The median beta-cell function (HOMA2-%B), (*p* < 0.01) insulin sensitivity (HOMA2-%S) (*p* < 0.01) and insulin resistance (HOMA2-IR) (*p* = 0.03) were also significantly different among the three points of data collection, suggesting a decrease in beta-cell function and an increase in peripheral insulin sensitivity. Proinsulin (*p* = 0.12) and C-peptide (*p* = 0.14) exhibited non-significant but trending changes, indicative of a decrease in insulin production. Detailed data are shown in Table 3. In the NG subgroup, only 2 patients (11.8%) maintained stable blood glucose levels within 12 months post-surgery, while 13 patients (76.5%) developed a prediabetic metabolic status and 2 patients (11.8%) developed NODM. Out of the two patients who developed postoperatively a diabetic metabolic condition, one patient showed insulin deficiency and was managed with insulin postoperatively. The second case, exhibiting a combined disorder involving both insulin deficiency and resistance, was treated with OHA.

**Table 3:** Impact of DP on blood glucose homeostasis in normoglycemic patients (*n* = 17)

Variable	Before surgery	3 Months	12 Months	*p*-Value
BMI kg/m^2^ (IQR)	23.4 (21.4–25.4)	22.3 (21.1–24.9)	23.5 (22.3–27.1)	< 0.07*
Fasting glucose mmol/L (IQR)	5.1 (4.87–5.35)	6.75 (5.78–7.55)	6.12 (5.18–9.59)	< 0.01*
Glucose (60 min) mmol/L (IQR)	7.36 (5.92–8.72)	5.27 (4.73–6.26)	8.55 (6.57–10.63)	< 0.001*
Glucose (120 min) mmol/L (IQR)	6.07 (5.39–8.49)	9.37 (8.15–10.83)	6.43 (5.97–8.35)	< 0.05*
HbA1c % (IQR)	5.3 (5.2–5.5)	7 (5.9–7.6)	7.2 (5.9–9)	< 0.01*
Insulin nmol/L (IQR)	0.07 (0.04–0.14)	0.04 (0.02–0.06)	0.06 (0.04–0.07)	< 0.001*
Insulin (60 min) nmol/L (IQR)	0.46 (0.27–0.75)	0.2 (0.12–0.35)	0.29 (0.19–0.46)	< 0.001*
Insulin (120 min) nmol/L (IQR)	0.2 (0.13–0.29)	0.3 (0.23–0.63)	0.29 (0.21–0.55)	0.09*
Proinsulin nmol/L (IQR)	1.6 (1–2.25)	1.6 (1–2.45)	7.1 (4–8)	0.12^#^
Proinsulin (60 min) nmol/L (IQR)	5.3 (4.25–9.6)	5.3 (4–13.65)	7.1 (4.3–10.95)	0.29^**#**^
Proinsulin (120 min) nmol/L (IQR)	11.6 (5.58–14.63)	7.05 (4.88–12.78)	7.4 (5.13–18.88)	0.08*
C-Peptide nmol/L (IQR)	0.62 (0.55–0.78)	0.54 (0.46–0.79)	0.59 (0.47–0.77)	0.14*
C-Peptide (60 min) nmol/L (IQR)	3.05 (2.28–3.78)	2.05 (1.68–2.58)	1.82 (1.48–2.61)	< 0.01*
C-Peptide (120 min) nmol/L (IQR)	1.98 (1.58–2.14)	1.95 (1.69–3.17)	2.45 (1.83–3.13)	0.13*
HOMA2-%B (IQR)	117.4 (101.1–135)	80.5 (54.3–102.1)	66.9 (49.7–102.1)	**< 0.01** ^**#**^
HOMA2-%S (IQR)	48.2 (33.4–66.9)	79.2 (48.7–95.6)	63.5 (49.8–86)	< 0.01*
HOMA2-IR (IQR)	2.07 (1.51–3)	1.26 (1.05–2.06)	1.57 (1.17–2.01)	**0.03** ^**#**^

* - The *p*-values in Table 3 refer to the comparison between preoperative and postoperative values after 3 months, # - the *p*-values reflect the comparison between preoperative and postoperative values after 12 months

Abbreviations: HOMA2 - updated Homeostasis Model Assessment; IQR - interquartile range

### Effects of DP on blood glucose homeostasis in patients with prediabetes

The analysis of HOMA indices indicated a slight decrease in HOMA2%B, with values of 119.2% (IQR: 93–150%) preoperatively, 107.3% (IQR: 84.5–119.3%) at 3 months postoperatively, and 91% (IQR: 58.3–118%) at 12 months postoperatively, with a *p*-value of 0.38. This was accompanied by a mild increase in insulin sensitivity, suggesting a compensatory response. Changes in median insulin (*p* < 0.01) and proinsulin (*p* < 0.05) were statistically significant. All other parameters were not significantly different between timepoints. However, trend changes indicative of insulin deficiency and efforts to compensate by increasing insulin sensitivity were observed. Detailed data are shown in Table 4.

**Table 4:** Impact of DP on blood glucose homeostasis in prediabetic patients (*n* = 22)

Variable	Before surgery	3 Months	12 Months	*p*-Value
BMI kg/m^2^ (IQR)	25.5 (23.8–28.1)	24.1 (21.8–26.9)	24.9 (22.4–26.2)	< 0.001*
Fasting glucose mmol/L (IQR)	5.45 (5.02–5.89)	6.1 (5.7–6.57)	5.99 (5.1–7.64)	0.33*
Glucose (60 min) mmol/L (IQR)	10.73 (8.67–12.77)	8.04 (6.33–9.32)	10.36 (8.56–12.47)	< 0.001*
Glucose (120 min) mmol/L (IQR)	9.81 (7.01–11.2)	10.82 (8.3–13.13)	7.54 (6.75–11.75)	0.07*
HbA1c % (IQR)	5.6 (5.5–5.9)	6.5 (6–8.3)	6.2 (5.7–7)	0.08*
Insulin nmol/L (IQR)	0.07 (0.06–0.1)	0.04 (0.03–0.08)	0.06 (0.04–0.07)	**< 0.01** ^**#**^
Insulin (60 min) nmol/L (IQR)	0.59 (0.37–0.91)	0.36 (0.24–0.79)	0.38 (0.23–0.45)	0.02*
Insulin (120 min) nmol/L (IQR)	0.33 (0.2–0.7)	0.45 (0.33–0.54)	0.38 (0.18–0.54)	0.49*
Proinsulin nmol/L (IQR)	2.65 (2–5)	2 (1–6.5)	1.55 (1–6)	**< 0.05** ^**#**^
Proinsulin (60 min) nmol/L (IQR)	12.2 (8.68–21.73)	19.9 (9.15–33.85)	7.6 (4.3–14.78)	0.11*
Proinsulin (120 min) nmol/L (IQR)	14.2 (7.33–29.73)	10.8 (6.6–19.1)	11.1 (10.2–26.05)	0.03*
C-Peptide nmol/L (IQR)	0.77 (0.62–0.97)	0.64 (0.49–0.76)	0.63 (0.5–0.84)	0.58*
C-Peptide (60 min) nmol/L (IQR)	3.61 (2.55–3.93)	3.47 (2.37–4.71)	2.33 (1.93–2.89)	**< 0.01** ^**#**^
C-Peptide (120 min) nmol/L (IQR)	2.96 (1.71–4.19)	2.39 (2.1–3.22)	2.91 (1.92–3.9)	0.26*
HOMA2-%B (IQR)	119.2 (93–150)	107.3 (84.5–119.3)	91 (58.3–118)	0.38*
HOMA2-%S (IQR)	56.4 (50.8–69.2)	63.9 (41.4–78.5)	64.9 (48.2–86.1)	0.76*
HOMA2-IR (IQR)	1.79 (1.58–1.97)	1.56 (1.27–1.97)	1.66 (1.34–2.09)	0.63*

* - the *p*-values in Table 4 refer to the comparison between preoperative and postoperative values after 3 months, # - the *p*-values reflect the comparison between preoperative and postoperative values after 12 months

Abbreviations: HOMA2, updated Homeostasis Model Assessment; IQR - interquartile range

The patients with prediabetes showed a similar trend as normoglycemic patients, with a slight BMI reduction in the first three months, followed by an increasing tendency over 12 months. In the IFG/IGT subgroup, only 33.3% of patients maintained stable blood glucose levels within 12 months post-surgery. Within 3 months, 57.1% of patients developed diabetic metabolic status, increasing to 60% within 12 months postoperative. Notably, only in one patient (4.6%) glucose homeostasis normalized during the follow-up period. Among patients in the IFG/IGT subgroup who developed postoperative DM (*n* = 12), the cause of diabetes was insulin deficiency and/or insulin resistance. Three months after surgery, the development of diabetes in 6 cases was attributed to the progression of pre-existing insulin resistance in the context of prediabetes, while insulin deficiency without insulin resistance occurred in 4 cases. Additionally, both factors were present in 2 cases. At the 12-month follow-up, further examination of the 9 diabetic patients revealed insulin deficiency as the primary cause of DM in 6 cases, insulin resistance in 2 cases and a combination of insulin deficiency and resistance in 1 case.

**Fig. 2:**
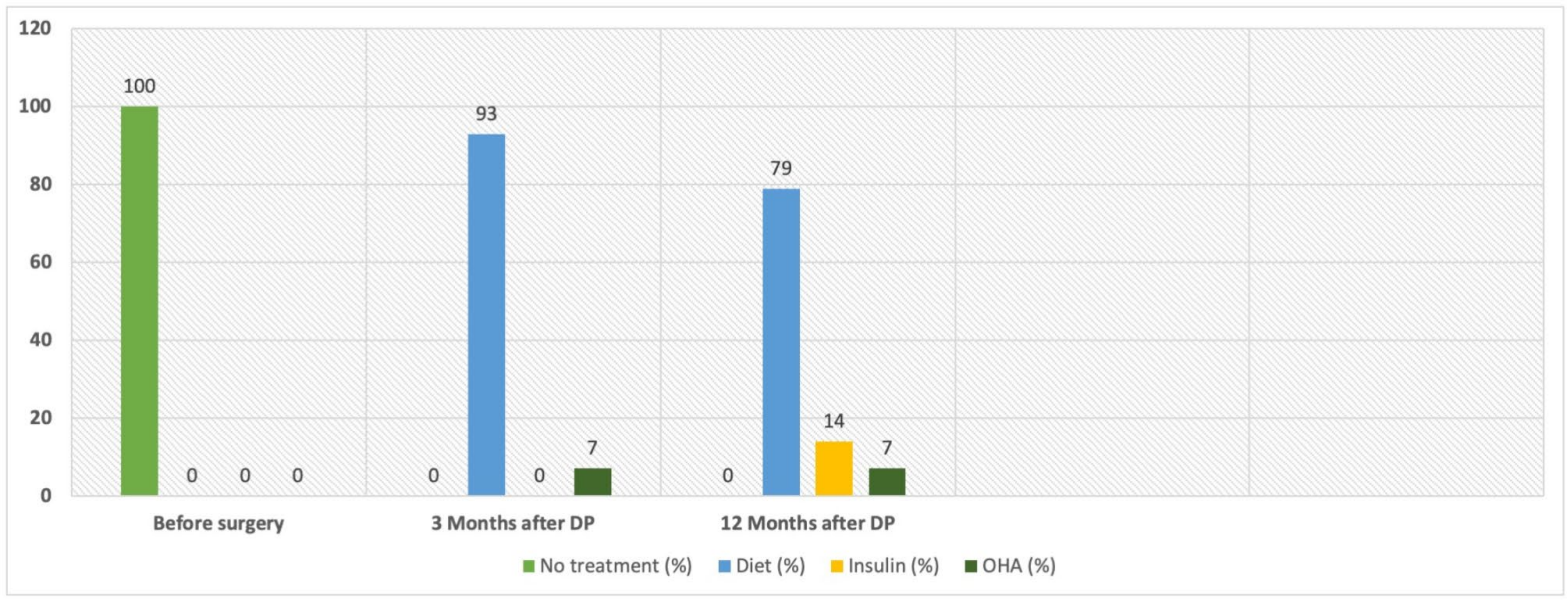
Postoperative diabetic therapy for subgroup characterized by IFG/IGT (*n* = 14) Abbreviations: DP - distal pancreatectomy; OHA - oral hypoglycemic agents

Out of the 14 patients who developed a NODM postoperatively, 2 (14%) were treated with insulin therapy, 1 (7%) with OHA, while the remaining 11 (79%) were managed with diet alone 12 months postoperatively (Fig. 2).

### Effects of DP on blood glucose homeostasis in patients with DM

The patients in the DM subgroup showed a stable glucose homeostasis over 12 months of postoperative follow-up. Among diabetic individuals, insulin concentration remained stable (*p* = 0.34) over the course of one year with a slight decrease observed in C-peptide (*p* = 0.27) and proinsulin (*p* = 0.22) levels. Analysis of HOMA indices revealed that insulin secretion and resistance remained almost unchanged. All the glucose metabolic parameters did not change significantly during the follow-up time. Out of 30 patients in this group, 1 patient improved his glycemic status to IGT. All other patients remained diabetic. All data are shown in Table 5. Patients with preoperative diabetes showed a statistically significant weight reduction at 3 months postoperatively, which continued at 12 months postoperatively.

**Table 5:** Impact of DP on blood glucose homeostasis in diabetic patients (*n* = 30)

Variable	Before surgery	3 Months	12 Months	*p*-Value
BMI kg/m^2^ (IQR)	27 (25.1–30.4)	26 (23.3–29)	24.3 (21.4–28)	< 0.001*
Fasting glucose mmol/L (IQR)	8.21 (7–11)	7.3 (5.93–8.17)	7.9 (5.8–12.41)	0.11*
HbA1c % (IQR)	7.4 (6.6–8.4)	6.2 (5.8–7.7)	6.8 (6.1–7.9)	0.26*
Insulin nmol/L (IQR)	0.07 (0.04–0.11)	0.07 (0.03–0.11)	0.07 (0.03–0.1)	0.34*
Proinsulin nmol/L (IQR)	4.65 (2.2–7)	3.9 (2.2–6.1)	2.8 (1.9–5.8)	0.22^#^
C-Peptide nmol/L (IQR)	0.89 (0.68–1.32)	0.66 (0.44–1.08)	0.74 (0.5–1.01)	0.27*
HOMA2-%B (IQR)	63.1 (41.8–91.1)	61.1 (40.9–86.6)	70.3 (35.9–95.8)	1*
HOMA2-%S (IQR)	50.2 (34.9–79.6)	46.5 (35.5–91.7)	48.4 (41.9–69.9)	1*
HOMA2-IR (IQR)	2 (1.25–2.86)	2.15 (1.09–2.82)	2.7 (1.44–2.39)	0.4^#^

# - the *p*-values in Table 5 refer to the comparison between preoperative and postoperative values after 3 months, * - the *p*-values reflect the comparison between preoperative and postoperative values after 12 months

Abbreviations: HOMA2, updated Homeostasis Model Assessment; IQR - interquartile range

Prior to surgery, DM was already diagnosed in a total of 30 patients. Six of these patients were treated with OHA before the procedure, of which 5 required a switch to insulin therapy postoperatively, while 1 patient continued to manage the condition adequately with OHA. Nine DM patients were managed with dietary measures alone before surgery. After the procedure, 6 of them required insulin therapy, 2 continued with diet alone, and 1 patient needed OHA.

**Fig. 3:**
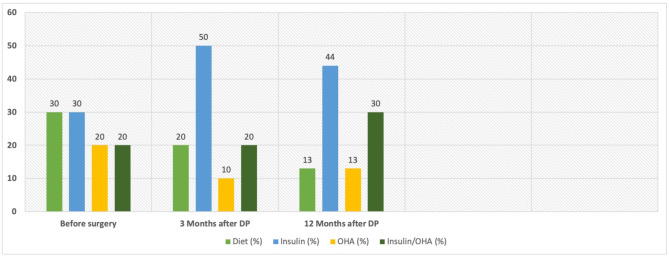
Postoperative diabetic therapy for subgroup with preoperative Diabetes mellitus (*n* = 30) Abbreviations: DP - distal pancreatectomy; OHA - oral hypoglycemic agents

Another group of 9 patients was insulin-dependent before the surgery and remained so afterward. In 5 of these cases, an increase in insulin dosage was necessary, while in 3 patients, no adjustment in their therapy was required. One patient continued on a combination of insulin and OHA postoperatively. Six patients with DM who had been on a combination therapy of OHA and insulin before surgery continued with the same regimen after the procedure. No further deterioration was observed in these patients, so no increase in dosage was needed (Fig. 3).

## Discussion

The incidence of NODM after DP exhibits considerable variability across the literature. This variability is attributed to multiple factors, including the diagnostic methodologies employed, the length of follow-up periods, concomitant diseases (such as obesity, cardiovascular disease or metabolic syndrome), the presence of CP and the underlying pathology. King et al. have documented that the incidence of NODM escalates progressively over time following DP [8]. In our study, the time-dependent postoperative interval revealed that 10–16% of NODM cases were identified exclusively through the OGTT, as neither glucose nor HbA1c levels were abnormal. Specifically, around 10% of patients were diagnosed using OGTT at 3 months postoperatively, and 16% at 12 months. This finding underscores the notion reported in the literature that the detected incidence rate of NODM post-DP is increased with the implementation of OGTT as a diagnostic tool [9]. The systematic review by Yu et al. identifies CP as a significant risk factor for NODM following DP [10].

Our study revealed that patients diagnosed preoperatively being prediabetic have a significantly increased risk (60%) of developing DM after DP compared to patients with preoperative NG, who exhibited an incidence rate of 11.7%. Prediabetes has been identified as a significant risk factor for the development of DM following pancreatic resections in various studies [9, 11]. The higher incidence of NODM in prediabetic patients compared to normoglycemic patients is likely due to both a reduction in insulin production and the absence of compensatory increases in insulin sensitivity. Prediabetic patients typically exhibit preoperative insulin resistance, which is unlikely to improve postoperatively, whereas normoglycemic patients tend to experience a compensatory increase in insulin sensitivity after surgery.

Recognizing prediabetes allows for proactive monitoring and potentially more targeted surgical and perioperative management [12]. A retrospective analysis of the impact of pancreatic head resection on blood glucose homeostasis in a cohort of patients with CP demonstrated similar trends, albeit without statistical significance [13]. This study suggests that pancreatic head resection does not have a more pronounced negative impact on glucose homeostasis. Notably, the patients studied by Hempel et al. underwent pancreatic head resection due to CP and were expected to have poorer residual pancreatic endocrine function. In contrast, a study conducted by Ehehalt et al. demonstrated that despite a reduction in beta-cell volume, an improvement in glucose homeostasis can occur following partial pancreatectomy [14]. This improvement was associated with the resolution of cholestasis. Additionally, normalization or reduction of blood glucose levels has been observed in some cases following PDAC resection, suggesting that the carcinoma itself may be a causative factor for NODM [15, 16].

Our results demonstrate a significant association between preoperative DM and PDAC, with preoperative DM diagnosed in 66.7% of patients with PDAC. This finding aligns with existing literature, which reports that approximately 45–65% of patients with PDAC present with preoperative DM [15, 17].

The results of our study suggest that the development of DM after DP is not solely attributable to insulin deficiency but often involves insulin resistance or insufficient compensatory increase in peripheral insulin sensitivity. In the literature, beta-cell loss due to pancreatic tissue resection is frequently cited as the primary cause of DM after pancreatic resection [5]. However, other contributing factors, such as insulin resistance, are only briefly mentioned and remain under-researched. This phenomenon following DP may also be coincidental. The decrease in insulin resistance despite reduced β-cell function can be explained by a compensatory increase in peripheral insulin sensitivity, as described in the literature following partial pancreatectomy. This adaptation likely helps mitigate the effects of reduced β-cell mass. However, in our study, this increase in insulin sensitivity was insufficient to restore glucose homeostasis or reduce the need for diabetes therapy. This may explain why normoglycemic patients develop postoperative diabetes less frequently than prediabetic patients, who may not experience the same compensatory increase in insulin sensitivity [18].

Our findings highlight the complex pathophysiology of DM after DP and underscore the need for differentiated therapeutic approaches based on the individual etiology of the disease. Despite this complexity, technological advances, particularly in telemonitoring, now offer effective patient monitoring and individualized management [19]. This is also playing an increasingly important role in diabetes treatment and blood glucose control through biosensors in smart wearable devices.

Numerous studies have indicated that patients are more likely to develop post-resection DM following DP compared to pancreatic head resection [20, 21]. The prevailing hypothesis suggests this may be directly related to the proportion of resected beta cells. Recently it has been shown that the density of beta cells across the length (head to tail) of the human pancreas is relatively uniform [22]. As DP typically involves the resection of a larger volume of pancreatic tissue, this should result in the loss of a greater number of beta cells.

In the literature, DM associated with pancreatectomy is often referred to as type 3c DM [23]. This term was initially introduced by the WHO in the Diabetes Classification of 1999 and accessed on April 2017 [4]. DM that occurs following pancreatic resection is often ambiguously classified in common classifications [3, 4]. However, with the advancement of pancreatic surgery, this condition is gaining increasing attention, and its prevalence is rising exponentially. A more precise subgrouping would be advisable. We would have suggested pancreatectomy-associated diabetes mellitus (PADM), but this abbreviation is already used in the literature for pancreatitis-associated DM [24]. Therefore, post-resectional diabetes mellitus (PRDM) could be a more suitable designation.

The present study has several limitations. First, its retrospective nature harbors the risk of a certain selection bias. The loss of some patients during the follow-up period further reduced the already small postoperative subgroups. Additionally, the follow-up period was limited to only 12 months, which restricts the ability to assess later effects of postoperative pancreatic resection on glucose homeostasis. Furthermore, the lack of determination of glucagon values in patients affected a complete assessment of islet cell function. The lack of quantitative analysis of pancreatic volume before and after surgery was due to the absence of standardized pre- and postoperative imaging, further limiting the understanding of the underlying mechanisms of PRDM. However, despite the small cohort size, the present study provides valuable insights considering beta-cell function and parameters of blood glucose metabolism within the three glycemic subgroups.

## Conclusion

Particularly in normoglycemic patients, changes in glucose metabolism can be observed shortly after surgery, even if they do not immediately develop DM. It is essential to preoperatively identify prediabetic patients and closely monitor them postoperatively, as they are at a significantly higher risk of developing DM after DP. The loss of pancreatic parenchyma and beta-cell mass leads to an early compromise in beta-cell function. Therefore, the indication for DP should be critically evaluated, especially in normoglycemic and prediabetic patients with a relative indication for surgery, while also considering the possibility of performing parenchyma-sparing resections.

## Data Availability

The data are available from the corresponding author on request.

## Abbreviations

ADA: American Diabetes Association
ASA: American Society of Anesthesiologists
BMI: Body mass index
CDC: Clavien-Dindo classification
CHD: Coronary heart disease
CHF: Chronic heart failure
CKD: Chronic kidney disease
CP: Chronic pancreatitis
DP: Distal pancreatectomy
HOMA2: Updated homeostasis model assessment
HTN: Arterial hypertension
IDDM: Insulin-dependent diabetes mellitus
IFG: Impaired fasting glucose
IGT: Impaired glucose tolerance
IPMN: Intraductal papillary mucinous neoplasm
MVR: Multivisceral resection
NET: Neuroendocrine tumor
NODM: New-onset diabetes mellitus
OHA: Oral hypoglycemic agents
OGTT: Oral glucose tolerance test
PD: Pancreaticoduodenectomy
PDAC: Pancreatic ductal adenocarcinoma
POPF: Postoperative pancreatic fistula
PPH: Postpancreatectomy hemorrhage
PRDM: Post-resectional diabetes mellitus
WHO: World Health Organization
